# Screening for Elevated Blood Lead Levels and Related Risk Factors among Thai Children Residing in a Fishing Community

**DOI:** 10.3390/toxics7040054

**Published:** 2019-10-12

**Authors:** Supabhorn Yimthiang, Donrawee Waeyang, Saruda Kuraeiad

**Affiliations:** 1 School of Public Health, Walailak University, Thaiburi, Thasala, Nakhon Si Thammarat 80160, Thailand; donrawee.wae@gmail.com; 2 School of Allied Health Sciences, Walailak University, Thaiburi, Thasala, Nakhon Si Thammarat 80160, Thailand; ksaruda@gmail.com

**Keywords:** blood lead level, boatyard, childhood, lead poisoning, fishing community, lead weights

## Abstract

The present study explored environmental and behavioral factors associated with elevated blood lead (Pb) levels in 311 children (151 girls and 160 boys), aged 3–7 years, who lived in a coastal fishing community of the Pakpoon Municipality, Nakhon Si Thammarat, Thailand. The geometric mean for blood Pb was 2.81 µg/dL, ranging between 0.03 and 26.40 µg/dL. The percentage of high blood Pb levels, defined as blood Pb ≥ 5 µg/dL, was 10.0% in boys and 13.9% in girls. Parental occupation in producing fishing nets with lead weights was associated with a marked increase in the prevalence odds ratio (POR) for high blood Pb (POR 17.54, 95%; CI: 7.093, 43.390; *p* < 0.001), while milk consumption was associated with 61% reduction in the POR for high blood Pb (POR 0.393, 95%; CI: 0.166, 0.931; *p* = 0.034). High blood Pb was associated with an increased risk for abnormal growth (POR 2.042, 95%; CI: 0.999, 4.174; *p* = 0.050). In contrast, milk consumption was associated with a 43% reduction in POR for abnormal growth (POR 0.573, 95%; CI: 0.337, 0.976; *p* = 0.040). After adjustment for age, the mean (standard error of mean, SE) values for blood Pb were 6.22 (0.50) μg/dL in boys and 6.72 (0.49) μg/dL in girls of parents with an occupation in making fishing nets with lead weights. These mean blood Pb values were respectively 2.3 and 2.5 times higher than similarly aged boys and girls of parents with other occupations. These data are essential for setting surveillance and programmes to prevent toxic Pb exposure, especially in children of coastal fishing communities in southern Thailand.

## 1. Introduction

Lead (Pb) is an environmental toxicant that causes serious harm to child health [1,2]. Children are highly susceptible to Pb toxicity due to hand-to-mouth behavior, high metal absorption rates, and the nervous system that is still in developing stage [3]. Pb enters the body through ingestion and breathing. It accumulates and causes toxicity in various tissues and organs that include the liver, kidneys, blood system, central nervous system (CNS), bone, and teeth [1]. Pb toxicity in the CNS cannot be restored to normal, and the World Health Organization considers mental retardation caused by excessive Pb exposure as one of the most serious environmental diseases [4]. There are no reports of blood Pb levels that are safe for children’s health. Moreover, chronic Pb exposure in childhood may predispose individuals to various diseases later in life.

Chronic exposure to Pb among children has been observed in various nations, including China, Brazil, Ukraine, South Africa, United States of America, and Australia [5,6,7,8,9,10,11,12]. Previous studies have suggested that the main reason for Pb exposure in children is environmentally related [5,6,7,8]. Elevation of blood Pb levels have been seen in children living in areas with high Pb contaminations, and residential areas have often been found to be a determinant of high blood Pb in children [6,7,8,9]. Children living near an electronic waste disposal area in China were found to have blood Pb levels between 4.14 to 37.78 µg/dL [10], while children lived near zinc and lead mining areas in Zambia had blood Pb levels ranging from 5.4 to 427.8 µg/dL [11]. In other studies, children living in fishing villages near the coast of South Africa and Tasajera (Colombian Caribbean coast) were found to have blood Pb levels ranging from 2.2 to 22.4 µg/dL and 0.4 to 50.1 μg/dL, respectively [8,12].

Nakhon Si Thammarat Province situates in the southern part of the Gulf of Thailand, where fishing communities with mini-scale repair boatyards exist, especially in Pakpoon suburb. In the traditional boat repair method, plumboplumbic oxide (Pb_3_O_4_) has been used; the strands of cotton ropes coated with Pb_3_O_4_ are caulked between wooden planks as waterproofing and to prevent barnacles. In one study, boat-repair workers were found to have blood Pb levels ranging from 9 to 89 µg/dL, and 67% of the workers had blood Pb levels exceeding 40 µg/dL, the level of concern for Pb exposure [13]. Other studies detected substantial amounts of Pb in soil and house dust from areas in close proximity to repair boatyards [14,15,16]. Of concern, mothers who made fishing nets with lead weights at home can introduce an additional Pb source to family members, especially young children who are the most vulnerable. Data of blood Pb levels in children in these communities are lacking. Hence, the present study was undertaken to assess the levels of environmental exposure to Pb among young children, 3–7 years of age, as reflected by blood Pb levels. We used blood Pb levels ≥ 5 µg/dL as a warning level, established by the U.S. Center for Disease Control [17]. In addition, we aimed to explore a range of environmental and behavioral factors, known as determinants of children’s blood Pb levels from the literature reports [5,6,7,8,9,10,11,12].

## 2. Materials and Methods

### 2.1. Study Design

The present study was in compliance with ethical standards. The Office of the Human Research Ethics Committee of Walailak University approved the study protocol (approval number 58/099, approval date 24 December 2015). The study was a community-based cross-sectional design that was undertaken from January 2016 to December 2018. Children, aged 3 to 7 years, were randomly chosen from the communities in Pakpoon suburb, where traditional wooden boat repairs were commonly practiced. The Taro Yamane equation was used to calculate the sample size, with a 5% level of significance and with a confidence coefficient of 95% [18]. The parents or guardians of all children provided written informed consent. We used structured interview questionnaires for information concerning a child’s age, gender, birth weight, body weight, and height, together with children’s behaviors including duration of outdoor play, home and school environment, diet, and health status.

### 2.2. Collection and Analysis of Blood Samples

The collection of children’s blood samples was performed by trained nurses. Approximately 3 ml of venous blood was collected from each child with ethylene diamine tetra-acetic acid as an anticoagulant. In preventing contamination during storage and transport, blood samples were stored at −20 °C in a sealed compartment. Blood samples were transported to Bangkok RIA Laboratory, Thailand for an assay for blood Pb levels with graphite furnace atomic absorption spectrophotometry. The limit of detection is 0.03 µg/dL.

### 2.3. Assessment of Child Growth

To assess growth of individual children, we used the standard weight for height curves for Thai children, prescribed by Thailand Ministry of Public Health [19]. Abnormal growth is defined as underweight or overweight using weight for height standards in accordance the Thai criteria; > +1.5 SD to > +3 SD (overweight), −1.5 SD to +1.5 SD (normal), < −1.5 SD to < −2 SD (underweight).

### 2.4. Statistical Analysis

We analyzed data with the SPSS software (SPSS Inc., Chicago, IL, USA). We examined the distributions of all continuous variables (age, body weight, height, body mass index [BMI]) for skewness. Data of the variables showing rightward skewing were presented as geometric mean ± standard deviation (SD) values. We used age-adjusted logistic regression analysis to derive the prevalence odds ratio (POR) for high blood Pb levels (≥ 5 μg/dL) and for abnormal growth. We used the generalized linear model (GLM) analysis to derive the age-adjusted mean blood Pb and age-adjusted BMI. We also used GLM to evaluate an effect of parental occupation and the child’s gender on blood Pb levels and BMI. *p* values ≤ 0.05 for two-tailed tests were assumed to identify statistical significance.

## 3. Results

### 3.1. Descriptive Characteristic of Study Children

A total of 311 children participated in the present study i.e., 160 were boys and 151 were girls. The average age was 4.67 years, ranging between 3 and 7 years (Table 1). The average body weight was 18.28 kg, the average height was 106 cm, and the average BMI was 16.6 kg/m^2^. Of 311 study children, 14.8% had low birth weight and 36.7% showed abnormal growth, based on Thailand Ministry of Public Health weight for height standards [19]. The geometric mean blood Pb level was 2.81 µg/dL, and 11.9% of children had blood Pb levels ≥5 µg/dL. The highest blood Pb level was 26.40 µg/dL, and 0.03 µg/dL was the lowest.

The environmental and behavioral data showed that 14.5% of children lived near repair boatyards, whereas 23.5% had parents with an occupation in producing fishing nets at home. More than half of children consumed milk (64.3%) and seafoods (53.7%). There were no statistically significant differences between boys and girls with respect to all parameters/factors considered.

### 3.2. Predictors of Blood Lead Levels ≥ 5μg/dL

To screen for potential risk factors for high blood Pb levels in study children, we used age-adjusted logistic regression analysis. Table 2 presents the results of a final model that incorporated ten independent variables: gender, milk consumption, seafood consumption, signs of Pb toxicity, painted toys, use of painted ceramics, peeling of paint chips, living near a repair boatyard, and parent occupation. Of these ten incorporated variables, only two variables, namely parental occupation and milk consumption, were associated with the prevalence odds ratio (POR) for high blood Pb. Parental occupation in producing fishing cast nets with lead weights was associated with 17.54 (95%; CI: 7.093, 43.39) fold increase in POR for blood Pb levels ≥ 5μg/dL, compared with all other occupations (*p* < 0.001). In contrast, a child’s milk consumption was associated with 61% reduction in the risk of having high blood Pb levels (POR = 0.393, 95%; CI: 0.166, 0.931; *p* = 0.034).

### 3.3. Predictors of Abnormal Growth

We used also age-adjusted logistic regression to determine potential effects of high blood Pb levels on children’s growth, defined as overweight or underweight in accordance with weight and height standards for Thai children. Table 3 presents the results of such analysis that incorporated seven independent categorical variables, including high blood Pb, milk and seafood consumptions, use of painted ceramics, living near a repair boatyard, and playing with painted toys. High blood Pb was associated with 2.042 (95%; CI: 0.999, 4.174) fold increase in POR for abnormal growth (*p* = 0.050). Seafood consumption was associated with 1.713 (95%; CI: 1.037, 2.831) fold increase in POR for abnormal growth (*p* = 0.036). In contrast, milk consumption was associated with 43% reduction in the risk of having abnormal growth (POR 0.573, 95%; CI: 0.337, 0.976; *p* = 0.040).

### 3.4. Effect-Size Estimate

**Figure 1:**
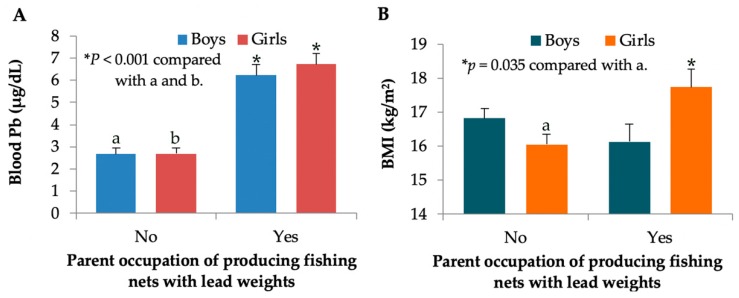
Blood Pb levels and BMI stratified by gender and parent occupation. Bars in (**A**) represent age-adjusted mean ± standard error of mean (SE) for blood Pb levels, while bars in (**B**) represent age-adjusted BMI ± SE in boys and girls from parents with and without an occupation of producing fishing nets with lead weights. * *p* values ≤ 0.05 identify statistical significance.

We next used generalized linear model (GLM) analysis to quantify effects of parental occupation on blood Pb levels and body mass index (BMI) of children. Figure 1A shows blood Pb levels in boys and girls of parents with and without an occupation in producing fishing nets with lead weights. Age-adjusted mean ± SE values for blood Pb in boys and girls of parents with the occupation of making fishing nets of 6.22 ± 0.50 and 6.72 ± 0.49 μg/dL were respectively 2.3 and 2.5 times higher than the same age-adjusted mean ± SE in boys (2.67 ± 0.27) and girls (2.68 ± 0.28) of parents with other occupations (*p* < 0.001 for boys and girls, Bonferroni test).

Of interest, Figure 1B indicated an effect of parental occupation on BMI in girls only. Age-adjusted mean ± SE values for BMI were 17.74 ± 0.53 kg/m^2^ in girls of parents with the occupation of producing fishing nets and 16.12 ± 0.54 kg/m^2^ in girls of parents with occupations other than making fishing nets (*p* < 0.035, Bonferroni test). On average, girls of parents with the occupation of producing fishing nets had a 9.1% higher BMI than the girls of parents of other occupations.

## 4. Discussion

**Figure 2:**
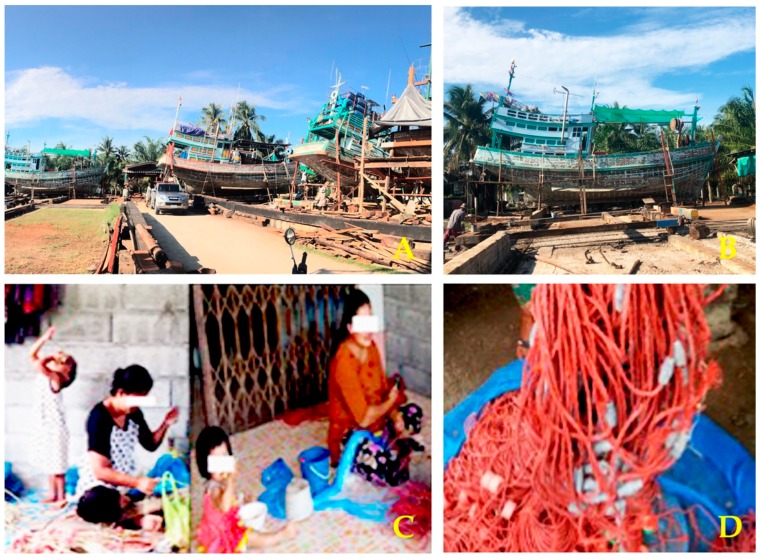
Boatyards, girls and working-at-home mothers, and a fishing net with lead weights. A total of 311 children were randomly selected from a fishing community in Pakpoon suburb, where repair boatyards existed (**A**,**B**). Girls were nearby while mothers were assembling lead weights to produce fishing nets (**C**). Approximately 180 lead weights are used to make a two-kg fishing net (**D**).

In the present study, we examined environmental exposure to Pb among 3–7 years old children, living in Pakpoon suburb, Nakhon Si Thammarat Province. The study children were randomly selected from a fishing community, where repair boatyards were located (Figure 2). The results showed that blood Pb levels ranged from 0.03 to 26.40 µg/dL. Although the average blood Pb level of 2.81 µg/dL was below the level of concern of ≥ 5 µg/dL, set by the CDC [17], 11.9% of children had elevated blood Pb levels ≥ 5 µg/dL. The blood Pb levels recorded for children in the present study appeared to be lower than the levels found in children living in fishing communities in the Tasajera and South Africa. Blood Pb levels in children in Tasajera, Colombian Caribbean coast, ranged from 0.4 to 50.1 μg/dL with 57.1% of study children having blood Pb levels ≥ 5 µg/dL [8]. Blood Pb levels in children of fishing communities in South Africa ranged from 2.2 to 22.4 µg/dL with 74% of study children having blood Pb levels > 5 µg/dL [12]. The 4.8 and 6.2 times higher percentages of high blood Pb in South Africa and Tasajera study might be due to different Pb exposure sources. The South Africa and Tasajera studies both found that living near a lead smelting area was strongly associated with children’s high blood Pb levels [8,12]. In contrast, there was no melting of Pb weights in the process of making fishing nets in this Thai study.

In the present study, we found that parent occupation involving lead weights was associated with 17.54 fold increase in risk of high blood Pb levels in children (*p* < 0.001). This might be attributable to environmental exposure via household Pb dust, water, and food contamination [20] from lead weights used in making fishing nets at home (Figure 2). However, living close to repair boatyards was not associated with high blood Pb levels (*p* = 0.872). Of note, blood Pb levels in both boys and girls of parents producing fishing nets were 2.3 and 2.5 times higher than similarly aged boys and girls of parents with other occupations (*p* < 0.001 for boys and girls). These data confirmed parents’ occupation as a strong determinant of blood Pb levels. The levels of Pb in dust, water, and food contamination in the households that used lead weights require a further study. Blood Pb levels in mothers should also be investigated since Pb is readily transported through the placenta.

A child’s milk consumption was associated with a 61% reduction in the risk for high blood Pb (*p* = 0.034) and a 43% reduction in the risk of having abnormal growth (*p* = 0.040). Milk has been reported to be a protective factor against Pb toxicity both in humans and experimental animals [21,22,23,24]. In an animal study, blood Pb levels decreased in lead-treated mice after nine weeks of daily milk intake. It is suggested that Pb absorption in gastrointestinal tract is reduced by the high calcium levels in milk, and that Pb absorption is enhanced by calcium deficiency [22,23]. In a longitudinal cohort study of children, aged 6–31 months [25], blood Pb levels were negatively correlated with calcium, magnesium, nickel, and zinc. It can be inferred that consumption of food rich in calcium and zinc can reduce Pb absorption. Another possible protective mechanism of milk might be due to organic substances that chelate Pb, thereby reducing Pb absorption and enhancing Pb excretion [21]. The synergism between Pb exposure levels and a lack of milk consumption is unknown. The present study indicated that milk is one of the factors that reduced the risk of high blood Pb levels in children. A quantitative study on milk intake is required.

Potential adverse effects of high blood Pb on children’s growth was observed; high blood Pb was associated with 2.04 fold increase in the POR for abnormal growth (*p* = 0.050), while seafood consumption was associated with a 1.71 fold increase in the POR for abnormal growth (*p* = 0.036). Likewise, blood Pb and seafood consumption were associated with decreased growth rates in other studies [25,26]. A negative effect of Pb on children’s growth may involve disruption of the endocrine system, causing circulating levels of insulin-like growth factor 1 to fall [27]. Pb may affect osteoblast and osteoclast development via 1, 25-dihydroxyvitamin D_3_ [28,29]. Exposure to Pb during childhood could affect growth in adolescents and adults. This was observed in a longitudinal study in Russia, where children with blood Pb levels ≥5 µg/dL showed the most height decrease at 12–15 years of age [30]. An association between seafood consumption and abnormal growth may be attributable to methymercury in seafood [31]. Thus, children with abnormal growth rates should be monitored. Further information should be sought to identify specific types of seafood and frequencies of consumption.

Interestingly, an effect of Pb on BMI increase was seen in girls only. This is a new finding. Gender-specific neurological effects of Pb have been seen in prenatal and preschool age exposure conditions [32,33,34]. Such gender specific effects of Pb may be caused by gene-specific DNA methylation patterns in the brain [33]. The gender-specific difference in BMI needs confirmation. Nevertheless, the association seen between children BMI and parent occupation could be used in growth prediction and obesity prevention programs in children.

In conclusion, the present study provided baseline data on environmental Pb exposure levels experienced by boys and girls, aged 3–7 years together with factors associated with the high blood Pb levels. These data are useful in setting Pb surveillance and Pb toxicity mitigation programs for children of fishing communities. Aspects of environmental Pb contamination need a further investigation. A Pb primary prevention program should be implemented in conjunction with a nutritional promotion campaign.

## Figures and Tables

**Table 1:** Descriptive characteristics of study children.

Parameters/Factors	Study Children			*p* Values
	All (*n* = 311)	Boys (*n* = 160)	Girls (*n* = 151)	
Age (years)	4.67 ± 1.14	4.67 ± 1.17	4.68 ± 1.11	0.776
Age range (years)	3−7	3−7	3−7	-
Body weight (kg)	18.28 ± 3.79	18.48 ±4.02	18.08 ±3.51	0.332
Height (cm)	106.0 ± 8.70	106.4 ± 9.50	105.6 ±7.80	0.466
Body mass index (kg/m^2^)	16.6 ± 3.30	16.7 ± 3.50	16.5 ± 3.00	0.540
Blood Pb (μg/dL)	2.81 ± 3.39	2.81 ± 3.37	2.80 ± 3.42	0.947
Range (μg/dL)	0.03−26.40	0.80−26.40	0.03−20.40	-
Prevalence rate (%)				
Blood Pb levels ≥ 5 μg/dL	11.9	10.0	13.9	0.287
Birth weight < 2500 g	14.8	12.5	17.2	0.241
Abnormal growth ^a^	36.7	36.9	36.4	0.934
Milk consumption	64.3	67.5	60.9	0.227
Seafood consumption	53.7	51.9	55.6	0.507
Living near repair boatyards	14.5	14.4	14.6	0.961
Parent occupation of producing fishing nets	23.5	22.5	24.5	0.677

Data for continuous variables are geometric means ± standard deviation (SD) values. ^a^ Abnormal growth is defined as underweight or overweight, based on the weight for height standard for Thai children; > +1.5 to > +3 SD (overweight), −1.5 SD to +1.5 SD (normal), < −1.5 SD to < −2 SD (underweight) (Nutrition Division Ministry of Public Health Thailand, 1999) [19]. *p* values ≤ 0.05 identify statistically significant differences between boys and girls. The Mann–Whitney U test was used to determine mean differences between boys and girls. The Chi-Square test was used to determine % differences between boy and girls.

**Table 2:** Predictors of blood lead levels ≥ 5μg/dL.

Independent Variables/Factors	Blood Pb Levels ≥ 5μg/dL				
	β Coefficients (SE)	POR	95% CI		*p* Value
			Lower	Upper	
Age (years)	−0.154 (0.194)	0.857	0.586	1.255	0.429
Gender (boy = 1, girl = 2)	0.470 (0.426)	1.599	0.694	3.685	0.270
Milk consumption	−0.934 (0.440)	0.393	0.166	0.931	0.034 *
Seafood consumption	−0.101 (0.436)	0.904	0.385	2.124	0.817
Symptoms of Pb toxicity	0.490 (0.431)	1.632	0.701	3.799	0.256
Painted toys	0.351 (0.459)	1.420	0.578	3.489	0.444
Use of painted ceramics	0.081 (0.508)	1.085	0.400	2.938	0.873
Peeling of paint chips	0.279 (0.498)	1.322	0.498	3.504	0.575
Living near repair boatyard	0.093 (0.581)	1.098	0.352	3.428	0.872
Parent occupation of fishing net production	2.865 (0.462)	17.54	7.093	43.39	<0.001 *

POR = Prevalence odds ratio. High blood Pb is defined as blood Pb levels ≥ 5μg/dL. The POR for high blood Pb was derived from logistic regression in which high blood Pb was a categorical dependent variable. Independent variables were listed in the first column. * *p* ≤ 0.05 identify the variable as a significant risk factor or predictor for high blood Pb levels.

**Table 3:** Predictors of abnormal growth.

Independent Variables/Factors	Abnormal Growth ^a^				
	β Coefficients (SE)	POR	95% CI		*p* Value
			Lower	Upper	
Age (years)	−0.084 (0.113)	0.920	0.737	1.148	0.459
Gender (boy = 1, girl = 2)	−0.116 (0.245)	0.891	0.551	1.440	0.637
Blood Pb levels ≥ 5 μg/dL	0.714 (0.365)	2.042	0.999	4.174	0.050 *
Milk consumption	−0.556 (0.271)	0.573	0.337	0.976	0.040 *
Seafood consumption	0.538 (0.256)	1.713	1.037	2.831	0.036 *
Use of painted ceramics	−0.552 (0.314)	0.576	0.311	1.066	0.079
Living near repair boatyards	0.561 (0.364)	1.753	0.860	3.574	0.123
Painted toys	0.277 (0.257)	1.319	0.798	2.181	0.281

POR = Prevalence odds ratio. ^a^ Abnormal growth is defined as underweight or overweight, based on weight for height standard for Thai children; > +1.5 SD to > +3 SD (overweight), −1.5 SD to +1.5 SD. (normal), < −1.5 SD to < −2 SD (underweight) (Nutrition Division Ministry of Public Health Thailand, 1999) [19]. * *p* ≤ 0.5 identify significant associations between abnormal growth and variables/factors listed in the first column.
